# Pegylated and liposomal doxorubicin is associated with high mortality and causes limited cardiotoxicity in mice

**DOI:** 10.1186/s13104-018-3260-6

**Published:** 2018-02-21

**Authors:** Christoffer Stark, Pekka Taimen, Timo Savunen, Juha Koskenvuo

**Affiliations:** 1 Research Center of Applied and Preventive Cardiovascular Medicine, University of Turku, Kiinamyllynkatu 10, 20520 Turku, Finland; 2 Department of Pathology, Turku University Hospital and University of Turku, Kiinamyllynkatu 4-8, 20520 Turku, Finland

**Keywords:** Pegylated and liposomal doxorubicin, Echocardiography, Myocardial fibrosis, Mortality, Thymosin beta 4

## Abstract

**Objective:**

We wanted to determine the impact of different doses of a pegylated and liposomal formulation of the cardiotoxic drug doxorubicin on cardiac function, fibrosis and survival in mice. The drug causes myocardial damage by producing reactive oxygen species, mitochondrial damage and lipid peroxidation. Thymosin beta 4 is a peptide with cardioprotective, anti-oxidant and anti-fibrotic properties and we further investigated whether the peptide could attenuate this drug-induced injury by measuring cardiac function and fibrosis.

**Results:**

Mice receiving high doses of doxorubicin died early during follow-up. Lowering the dose improved survival but did not markedly impair cardiac function on echocardiography and caused only limited fibrosis on histology. Thymosin beta 4 had only a mild protective effect on early cardiac function and did not significantly influence myocardial fibrosis. In conclusion, the use of pegylated and liposomal doxorubicin was not appropriate for inducing experimental cardiomyopathy. Thymosin beta 4 therapy was not beneficial in this setting.

## Introduction

Doxorubicin is a widely used anti-cancer drug that belongs to the anthracycline family. It interferes with DNA and RNA synthesis by binding to the double-stranded DNA helix, thus preventing replication. The drug has various short and long-term cardiotoxic effects. Acute myocardial depression is observed within the first week of treatment and is considered reversible. Chronic effects can lead to dilated or restrictive cardiomyopathy and progressive heart failure [1]. The cardiotoxic effects are dose-dependent. Pegylated and liposomal formulations of doxorubicin (PLD) are generally better tolerated and causes less cardiotoxicity [1]. These formulations have a significantly longer half-life in the circulation, which leads to lower peak concentrations. The distribution of pegylated liposomal doxorubicin to the myocardium is also lower [2]. The deleterious effects of anthracyclines on the myocardium are mainly related to the production of reactive oxygen species, mitochondrial damage and lipid peroxidation of the myocardial cell membrane [1–3]. These effects can lead to myocardial necrosis or activation of Caspase 3-induced apoptosis. Doxorubicin is also associated with a decreased production of protective anti-oxidant enzymes and increased inflammatory mediators such as interleukins 1β and 6 and tumor necrosis factor alpha (TNF-α) [3, 4]. In mouse animal models, doxorubicin treatment has been shown to reduce cardiac function and lead to dilatation and fibrosis of the left ventricle. Functional deterioration of the left ventricle has been observed after single or cumulative doses of 5–25 mg/kg. Mortality rates with similar doses have ranged from 10 to 38% [4–8]. Thymosin beta 4 (TB4) is a peptide with cardioprotective properties [9]. In vitro studies have demonstrated that it reduces the formation of ROS in cardiomyocytes and cardiac fibroblasts and increases the expression of anti-oxidative enzymes. The peptide is also related to a reduction in Caspase 3, TNF-α and interleukin-6 in vivo [10, 11]. In this study, we wanted to determine the influence of different doses of PLD on cardiac function, myocardial fibrosis and survival. We also studied the influence of TB4 treatment on these parameters as several of the known injury mechanisms of doxorubicin are therapeutic targets of the peptide.

## Main text

### Materials and methods

For the study 42 male FVB/n mice (Central animal laboratory of the University of Turku) weighing 26–30 g were used. The animals were divided into three groups with different PLD dosing protocols and follow-up times. All three study groups were divided into controls and TB4 therapy groups. Group 1 received a single intraperitoneal (i.p.) injection of PLD 20 mg/kg suspended 1:1 in saline (Caelyx 2 mg/ml, Janssen-Cilag OY, Espoo, Finland). TB4 (Genway Biotech, San Diego, CA, USA, Catalog No: GWB-DD4CD5) was administered i.p. (6 mg/kg suspended in 100 µl saline) daily for 14 days (n = 5). Control animals received plain saline (n = 5). The animals in group 2 received a single i.p. injection of PLD 10 mg/kg and the treatment group received five doses of TB4 i.p. (6 mg/kg) every third day (n = 5). Plain saline was administered to controls (n = 5). In group 3 the animals were given four weekly i.p. injections of PLD 5 mg/kg. TB4 (6 mg/kg) was given daily for 28 days by i.p. injection to treated animals (n = 11), while control animals received plain saline (n = 11). Animals were housed in individual cages with a 12:12 h dark–light cycle and had ad libitum access to food pellets and water. At the end of follow-up, euthanasia was performed by cervical dislocation after CO_2_ asphyxiation.

The animals in group 3 underwent echocardiography 10, 20 and 35 days after the first PLD injection by an investigator blinded to group assignments. For imaging the animals were pre-anesthetized with 5% isoflurane (Forane, Baxter Healthcare Corp, Deerfield, IL, USA) and placed on a heating pad. The animals were allowed, to breathe spontaneously and anesthesia was maintained using 1.5% isoflurane administered through a nasal mask. Echocardiography was performed using a high-frequency small animal imaging platform (Vevo 2100, FUJIFILM VisualSonics Inc, Toronto, Ontario, Canada). Parasternal long-axis images and M-mode images were obtained and analyzed for measuring left ventricle (LV) fractional shortening (FS), systolic (LVIDs) and diastolic internal diameters (LVIDd).

Heart tissue samples from animals in groups 2 and 3 were collected after euthanasia. The samples were formalin fixed, paraffin-embedded and transferred to glass-slides for Van Gieson staining. The left ventricle was analyzed for interstitial fibrosis in a blinded fashion by measuring the stained area in five randomly selected high-power (×40) fields. ImageJ software was used for image analysis.

Kaplan–Meier plots were used for survival analysis. Unpaired two-sided t tests were performed for comparative analysis between treated animals and controls in the echocardiography and histological analyses. 2-way ANOVA analysis was used for comparing different time-points. A p value of less than 0.05 was considered significant.

### Results

**Fig. 1:**
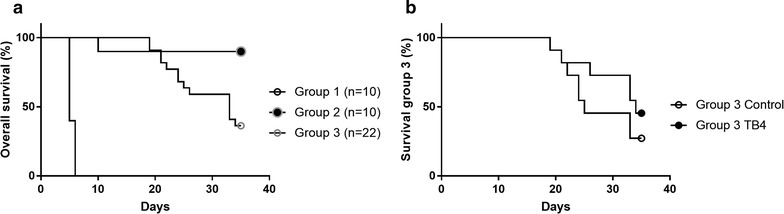
Overall combined survival (%) in the three PLD groups (**a**). Group 1 received a single dose of PLD 20 mg/kg, group 2 a single dose of 10 mg/kg and group 3 four weekly injections of 5 mg/kg. Control animals (saline) and TB4 treated animals (6 mg/kg daily for 28 days) in group 3 had similar mortality rates (p = 0.27) (**b**)

All animals in group 1 died during the first week of follow-up and no further analyses were performed. The deaths were considered related to PLD toxicity as the animals showed anorexia and fatigue. In group 2 only one animal died after treatment initiation. The remaining animals were euthanized after 10 weeks and the hearts excised for histological analysis. In group 3 the mortality rate increased after the third doxorubicin injection and only 8/22 animals completed the 5-week follow-up. Therefore a cumulative doxorubicin dose of 15–20 mg/kg seemed to be lethal (Fig. 1a). Kaplan–Meier analysis showed no difference in mortality between TB4 treated animals and controls in group 3 (p = 0.27) (Fig. 1b). There was a continuous decrease in body weight between day 1 and day 35 in both controls and treated animals (− 2.9 g vs − 3.1 g, p = 0.95). No animals were excluded from the survival analysis.

**Fig. 2:**
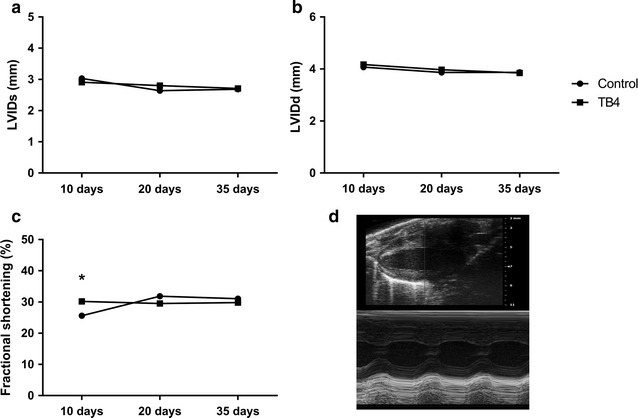
LVIDs (**a**) and LVIDd (**b**) and FS (**c**) for controls and TB4 treated animals in group 3. The animals received 4 weekly i.p. injections of PLD (5 mg/kg) and TB4 (6 mg/kg) or saline daily (means). Representative echocardiography image of the left ventricle (**d**). *p<0.05, all other differences = NS

All surviving animals in group 3 underwent serial echocardiography 10, 21 and 35 days after the first PLD injection. FS decreased more in control animals at day 10 (26 ± 4% versus 30 ± 1%, p < 0.05) but returned to similar levels as for TB4 treated animals at days 21 and 35 (31.9 ± 3.6% vs 29.5 ± 3.5%, p = 0.17 and 31.1 ± 6.1% vs 29.8 ± 8.1%, p = 0.86). No differences in LVIDs at 10 days (3.03 ± 0.24 mm vs 2.91 ± 0.15 mm, p = 0.18), 21 days (2.64 ± 0.42 mm vs 2.80 ± 0.27 mm, p = 0.36) or 35 days (2.69 ± 0.56 mm vs 2.71 ± 0.57 mm, p = 0.96) was observed. LVIDd at 10 days (4.07 ± 0.20 vs 4.17 ± 0.21, p = 0.27), 21 days (3.87 ± 0.54 vs 3.97 ± 0.30, p = 0.60) and 35 days (3.87 ± 0.47 vs 3.85 ± 0.51, p = 0.95) were also similar. In controls the decrease in LVIDs and the increase in FS were statistically significant between days 10 and 21 (p < 0.05). There were no significant differences between other time points (Fig. 2).

**Fig. 3:**
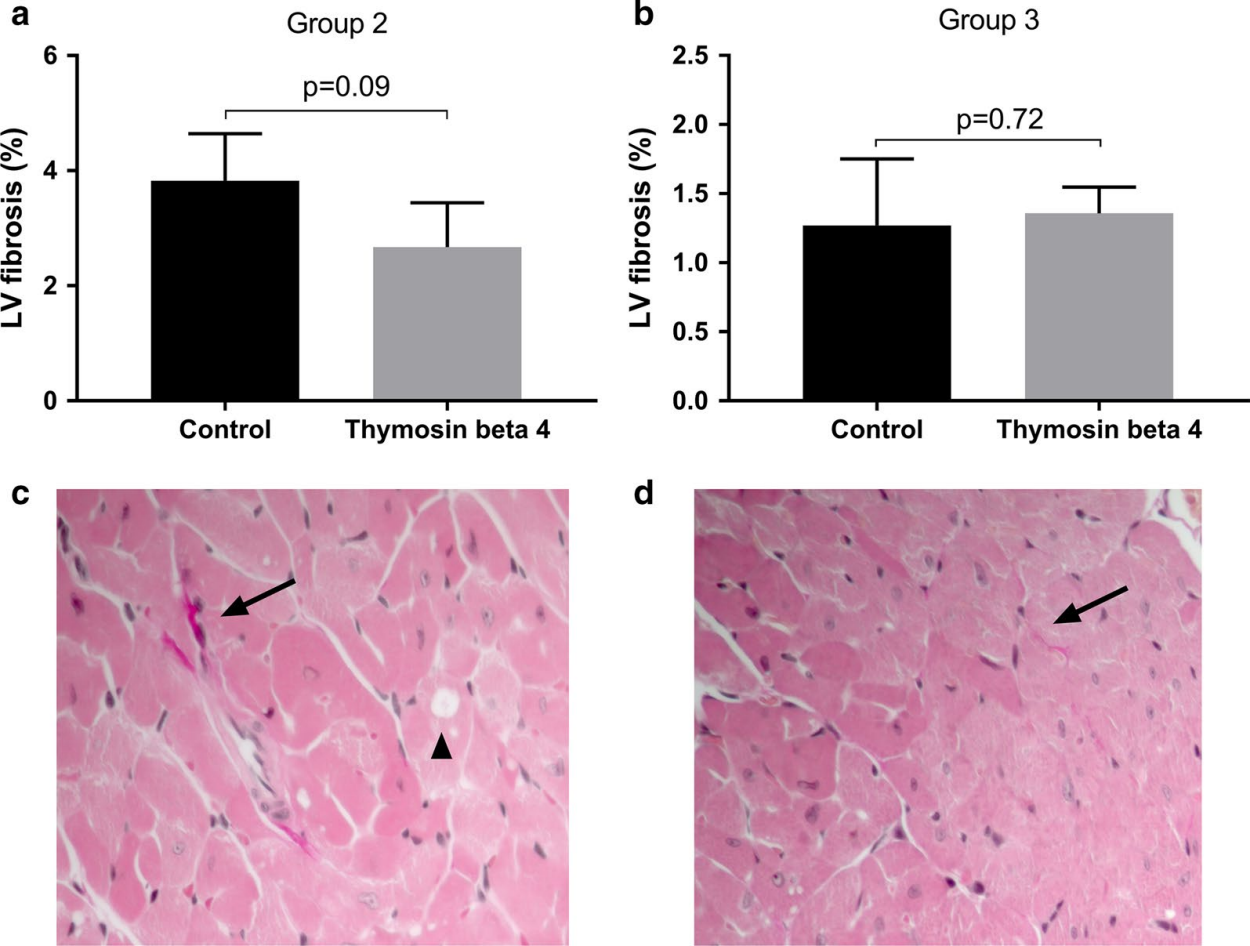
Myocardial fibrosis in study groups 2 (**a**) and 3 (**b**) (mean ± SD). Histology with Van-Gieson stain in representative samples in group 2 (**c**) and 3 (**d**). Arrows shows fibrosis and arrowheads vacuolization of cardiomyocyte cytoplasm

Tissue samples from the left ventricle were analyzed for myocardial fibrosis in all surviving animals in groups 2 and 3, 10 and 5 weeks after treatment initiation, respectively. At 5 weeks, the amount of fibrosis was similar between controls and TB4 treated animals (1.27 ± 0.48% vs 1.36 ± 0.19%, p = 0.72) (Fig. 3b). The total amount of myocardial fibrosis increased by week 10 but the differences between controls and TB4 therapy remained statistically insignificant (3.83 ± 0.82% vs 2.67 ± 0.77%, p = 0.09) (Fig. 3a). The increase in fibrosis between 5 and 10 weeks was statistically significant within controls and TB4 treated animals (< 0.05) but not between groups (p = 0.99 and p = 0.14). In group 2 some vacuolization of cardiomyocyte sarcoplasm was observed, probably due to PLD cardiotoxicity.

### Discussion

The mortality rates in this study were higher than expected and we did not observe any influence of TB4 therapy on survival. Mortality increased when the PLD dose exceeded 15 mg/kg. The dosing protocol in group 3 was chosen based on the observations in groups 1 and 2 with the aim to increase survival and to also maximize PLD cardiotoxicity. In this group TB4 was administered daily, in order to achieve better theoretic cardioprotection. The mortality rates in previous trials with doxorubicin doses of 15–25 mg/kg have been significantly lower during the first 10 weeks of follow-up (10–38%) [3, 5]. Later mortality, beyond week 10 on the other hand is reported to be > 75% with the same dosage [6, 7]. In a recent study, PLD 20 mg/kg was used as a single intraperitoneal injection but the authors did not report mortality rates [12]. In our study left ventricle chamber diameters and FS remained constant between days 10 and 35 although mortality exceeded 50%. The more common clinical adverse effects associated with Caelyx compared to plain doxorubicin are gastrointestinal disorders. Bone marrow suppression is also possible, although severe depression is uncommon in clinical scenarios [2]. The animals in this study suffered from mild anorexia as seen by the failure to gain weight during the follow-up period. This could indicate that the animals died from other than heart failure related causes.

On echocardiography left ventricular fractional shortening was slightly higher in TB4 treated animals at 10 days after the first PLD dose. The absolute change was however minimal and at later time-points the difference disappeared. It is unlikely that this finding has any practical significance but might indicate a mild protective effect of TB4 on acute myocardial depression. In a previous report using a similar animal model, PLD had no influence on left ventricle ejection fraction and there was only a mild increase in ventricular dimensions indicating limited cardiotoxicity [12].

The amount of myocardial fibrosis was higher at 10 weeks after PLD administration although the animals only received half the cumulative dose (10 mg/kg versus 20 mg/kg) compared to the group studied at 5 weeks. This could indicate that myocardial fibrosis is a time-dependent process rather than just related to PLD dose. TB4 did not significantly impact the amount of fibrosis, although it has previously been associated with a reduction in myocardial fibrosis after ischemic heart injury [11].

### Conclusions

The use of PLD did not seem to be appropriate for inducing cardiomyopathy in this mouse model as no substantial impairment of cardiac function was documented and the treatment was associated with high overall mortality. The treatment caused some myocardial fibrosis but its impact on cardiac function is unclear. In this setting, TB4 did not significantly influence survival nor cardiac function after the acute phase or the amount of cardiac fibrosis. Our results are mainly negative (or neutral) and the animal model was not optimal for studying TB4 mediated cardioprotection. Based on previous reports we thought the model would induce are stronger cardiomyopathy. We tried to overcome this issue by increasing the PLD dose but ended up with increased mortality. This finding is however a result and might be valuable with regard to future experiments.

## Limitations

The unexpected high mortality caused variability in the dosing protocols which impacts comparison of the groups. Based on the echocardiographic findings, PLD administration was not severely cardiotoxic and therefore any possible therapeutic effect of TB4 in the chronic phase is possibly unrecognizable.

## Abbreviations

PLD: pegylated and liposomal doxorubicin
TNF-α: tumor necrosis factor alpha
TB4: thymosin beta 4
i.p.: intraperitoneal
FS: fractional shortening
LVIDs: left ventricle internal diameter during systole
LVIDd: left ventricle internal diameter during diastole
